# AC-Electroosmosis-Assisted Surface Plasmon Resonance Sensing for Enhancing Protein Signals with a Simple Kretschmann Configuration

**DOI:** 10.3390/s22030854

**Published:** 2022-01-23

**Authors:** Kyohei Terao, Shohei Kondo

**Affiliations:** Department of Intelligent Mechanical Systems Engineering, Kagawa University, Takamatsu-shi 761-0396, Japan; s.kondou0920@gmail.com

**Keywords:** surface plasmon resonance sensing, AC electroosmosis, protein detection

## Abstract

A surface plasmon resonance (SPR) sensor chip fabricated with a comb-shaped microelectrode array to supply alternating current (AC) voltage is reported. The chip induces circulating flow near the surface (i.e., AC electroosmosis). The circulating flow provides a mixing effect, which enhances the binding of the analyte molecules. We evaluated the SPR characteristics of the chip and demonstrated an improvement in protein binding to the chip surface. SPR sensor chips with comb-shaped microelectrodes were fabricated using standard UV lithography. Sensing experiments were conducted using a standard Kretschmann-type SPR measurement system. To demonstrate the mixing effect of AC electroosmosis, we evaluated the binding of immunoglobulin G molecules onto the sensor surface where anti-immunoglobulin G antibodies were covalently immobilized. The result indicates that the amount of binding increases by a factor of 1.7 above that achieved by using a conventional chip, suggesting enhancement of the protein signal.

## 1. Introduction

Surface plasmon resonance (SPR) sensing offers advantages in terms of label-free and real-time techniques with high sensitivity [1,2,3,4]. SPR sensors are powerful tools in the fields of biochemistry, clinical diagnosis, food analysis, and environmental biology [5,6]. Among the several types of SPR sensing techniques, the Kretschmann-type configuration is the most widely used because of its simple optical setup and sensor chip geometry [7,8], and the systems are commercially available (e.g., Biacore). Improvement of its detection limit and integration of various functions on a chip has been reported [9,10,11] and is expected to broaden its potential applications. To expand the detection limit for small and low-concentration proteins, we have developed a Kretschmann-type sensor chip that enhances the binding of analyte protein molecules onto the sensor surface using an electrokinetic phenomenon, alternating current electroosmosis (ACEO).

In the Kretschmann configuration, a sensor chip with a flat metal layer on a transparent substrate (typically a gold layer on a glass substrate) is placed on a prism, where incident light is totally reflected, exciting surface plasmon waves on the metal surface only at a specific incident angle (the SPR angle). The intensity of the reflected light is lowest at the SPR angle, which depends on the refractive index of the medium in contact with the metal. Analyte proteins in solution bind to the ligands on the sensor chip, changing the refractive index near the chip surface; these changes enable the amount of ligand binding to be measured. This means that increasing the number of analyte molecules bound to the sensor surface enhances the sensor signal.

ACEO is an electrokinetic phenomenon induced by the application of AC voltage to microelectrodes, causing circulating flow above the electrode surface that mixes the analyte solution near the surface and increases the chance of analyte molecules binding to the ligands on the surface [12,13,14,15,16]. The use of electrokinetic phenomena has been reported in the literature [17,18]; however, it requires a complex optical system for imaging sensor chips designed for SPR imaging [19,20], and the improvement of protein binding has not been proven with a sensing setup without imaging function. To broaden the application of electrokinetic effects in SPR sensing, the verification of the efficiency of ACEO in protein detection is reported here using a standard Kretchmann-type SPR sensing setup.

**Figure 1:**
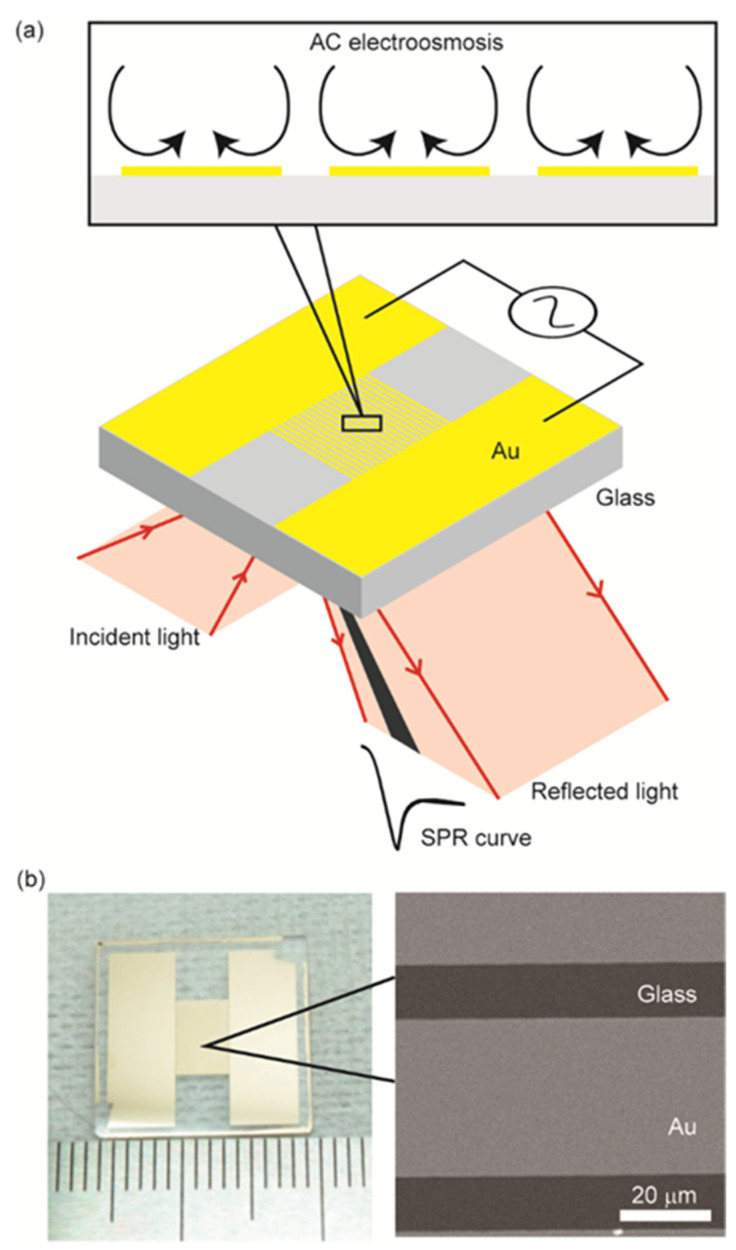
SPR sensing with a comb-shape Au chip for ACEO: (**a**) Schematic of SPR sensing with the Kretschmann configuration; the inset shows the side view of the flow induced by ACEO above the microelectrodes; (**b**) Sensor chip of Au comb-shape microelectrodes.

We developed a sensor chip with comb-shaped Au microelectrodes that induces ACEO in the solution in contact with the chip surface and functions as the SPR sensing basis simultaneously (Figure 1a). The microelectrodes were aligned in the direction of the incident light to prevent blocking of the propagation of surface plasmon waves. The sensor chip is readily adapted to the standard Kretschmann-type SPR sensing systems, which are commercially available without changing their optical setup. In this study, we evaluated the SPR performance in ACEO applications and demonstrated the enhancement of protein binding using an antibody-immobilized sensor surface.

## 2. Materials and Methods

### 2.1. Sensor Chip Fabrication

The sensor chip had two rectangular Au areas and comb-shaped microelectrodes between them. The microelectrodes had a width of 60 μm with a gap of 20 μm (Figure 1b). Glass chips (BK7, NTT-AT Corp, Tokyo, Japan) with a refractive index of 1.515 and measuring 16 mm × 16 mm × 1 mm were cleaned with a piranha solution (H_2_SO_4_:H_2_O_2_ = 3:1) for 10 min at 85 °C and washed three times with pure water. A 5-nm-thick Cr layer was formed on the chip by using a thermal deposition system (VPC-1100, ULVAC Corp, Kanagawa, Japan) to act as an adhesive layer; this was followed by the deposition of a 45-nm-thick Au layer. The photoresist AZ-1505 (Merck KGaA, Darmstadt, Germany) was coated on the Au layer and patterned using a maskless lithography system (MX-1204, Japan Science Engineering Co., Kyoto, Japan). The Au layer was then patterned by wet etching with a KI3 solution. Subsequently, the Cr layer was removed by using a Cr etchant. The AZ-1505 pattern was removed using the AZ remover. Prior to SPR sensing, the chip was treated with a UV-ozone cleaner (UV253H, Filgen Inc., Nagoya, Japan).

### 2.2. Sensing Operation

We used a Kretschmann-type SPR sensing system (Smart-SPR, NTT-AT Corp., Tokyo, Japan) with a measurement range from 65° to 75° and an area of 4.8 mm × 2 mm of the chip surface and a 770 nm light source. An SPR sensor chip was placed on the prism, where a polydimethylsiloxane block having two through holes was attached to the sensor surface to form two wells for sample application: one for the detection of the sample solution and the other for a reference to compensate for thermal drift. Solution exchange was conducted by pipetting from the top of the polydimethylsiloxane wells for batch-type operation. The AC voltage of the sinusoidal wave was applied to the microelectrodes using a function generator (WF1974, NF Corp., Yokohama, Japan) to induce ACEO.

### 2.3. ACEO Evaluation

Circulating flow on the sensor surface was visualized with fluorescent microbeads (green fluorescent polymer microspheres with a diameter of 1.9 μm, Bangs Laboratories. Inc., Fishers, United States) in a 10 mM phosphate-buffered saline (PBS). The movement of the beads was observed using a fluorescence microscope (BX-53, Olympus Corp., Tokyo, Japan). We applied AC voltages of various amplitudes and frequencies and checked the damage and dysfunction caused by the electrode reactions.

### 2.4. Protein Detaction

Protein detection was performed using anti-immunoglobulin G (anti-IgG) antibodies and IgG molecules as ligands and analytes, respectively. The reagents used in the experiments were IgG (Sigma-Aldrich Co., St. Louis, United States), anti-IgG (G, Sigma-Aldrich Co., St. Louis, United States), PBS (Biacore, Uppsala, Sweden), N-hydroxysuccinimide (NHS) (FUJIFILM Wako Pure Chemical Corp., Osaka, Japan), 1-ethyl-3-(3-dimethylaminopropyl) carbodiimide hydrochloride (EDC) (Tokyo Chemical Industry Corp., Tokyo, Japan), ethanolamine (Biacore, Uppsala, Sweden), and 4,4′-dithiodibutyric acid (Dojindo Laboratories, Kumamoto, Japan). A 10 nM 4,4´-dithiodibutyric acid–PBS solution was dispensed onto an SPR sensor chip and incubated for 12 h to form a carboxy-terminated self-assembled monolayer on the Au layer. Then, the chip was placed on the SPR sensing system, where a 0.1 M NHS and 0.05 M EDC–PBS solution was dispensed and incubated for 10 min to activate the sensor surface. After that, anti-IgG antibodies were covalently immobilized on the sensor surface as ligands by applying a 50 μg/mL anti-IgG–PBS solution and incubating for 10 min. To prevent nonspecific binding, we dispensed a 1 M ethanolamine–PBS solution that blocks the unreacted groups. A 0.5 mg/mL IgG–PBS solution was applied as an analyte solution and incubated for 10 min. All processes were conducted at room temperature. Between each step, the sensor chip was washed with a PBS solution. We obtained the time course of the SPR angle (SPR sensorgram) during these steps: The signals of these steps in the detection channel and of a PBS solution in the reference channel were recorded, and we defined the sensor signal by subtracting the reference signal from the detection signal.

## 3. Results and Discussion

### 3.1. ACEO on the Sensor Chip

The sensing area was partially covered with Au comb-shaped microelectrodes. The Au area propagates surface plasmon waves for SPR sensing, whereas the glass area (i.e., electrode gap) does not. Therefore, the reduction in the Au area increases the reflected light intensity at the SPR angle, preventing us from obtaining a stable SPR angle for sensing. In addition, the flow induced by the ACEO depends on the strength of the electric field, which is defined by the electrode gap. From these conditions, we set the width of the microelectrodes and the gaps to be 60 and 20 μm (Au:glass = 3:1), respectively. 

**Figure 2:**
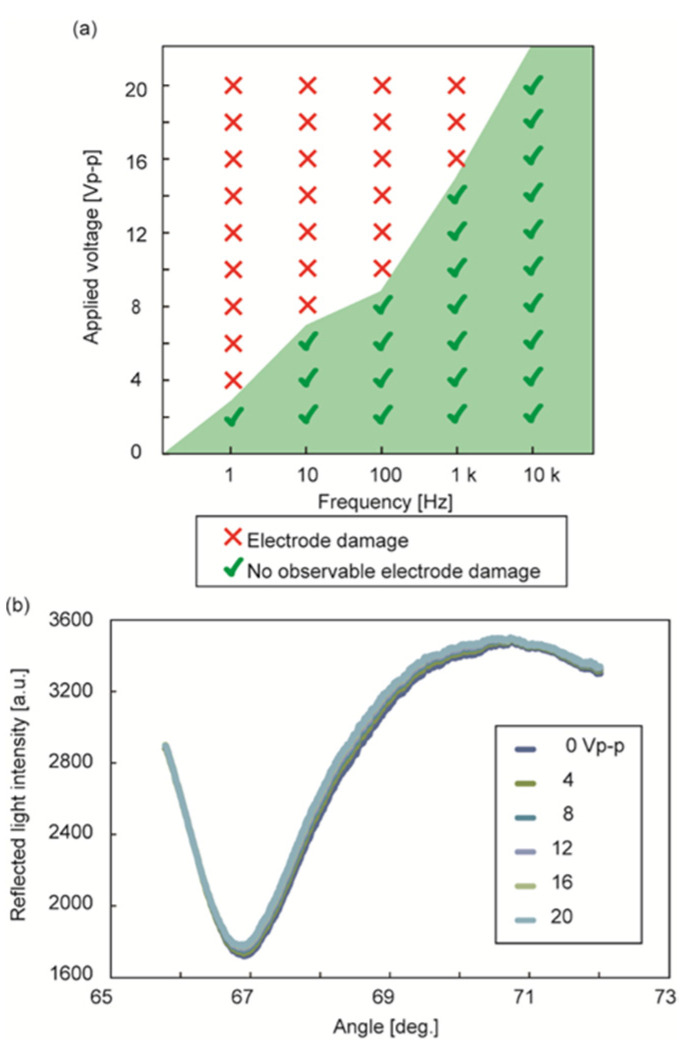
Effects of sinusoidal voltage application to microelectrodes: (**a**) Electrode damage caused by voltage application of various amplitudes and frequencies in 10 mM PBS solution; (**b**) SPR curves in voltage application (0–20 V_p-p_ and 10 kHz).

Because the application of voltage may cause electrode reactions, resulting in electrode dysfunction, we investigated the range of the amplitudes and frequencies applicable to the sensor chip using 10 mM PBS as a working solution (Figure 2a). The results indicated that the green region in the figure shows operable voltage conditions, and higher frequencies inhibit electrode reactions to allow higher voltage. We evaluated the SPR curves obtained in the peak-to-peak voltage range of 0 to 20 V_p-p_ (with an electric field strength between electrodes of 20 MV/m) at a frequency of 10 kHz (Figure 2b). The results revealed that the SPR curves are not significantly different from those without voltage application, which is consistent with the literature results [19,21]. The voltage applied to the Au layer causes an SPR angle shift at the area [22]. The SPR curve in our setup, however, is obtained through the sum of the light reflected at the electrodes charged positively and negatively; therefore, the shift can be cancelled. Furthermore, the voltage frequency (10 kHz) is much higher than the sampling rate of the SPR angle (1 Hz), averaging the effect of voltage application on the SPR angle shift. 

**Figure 3:**
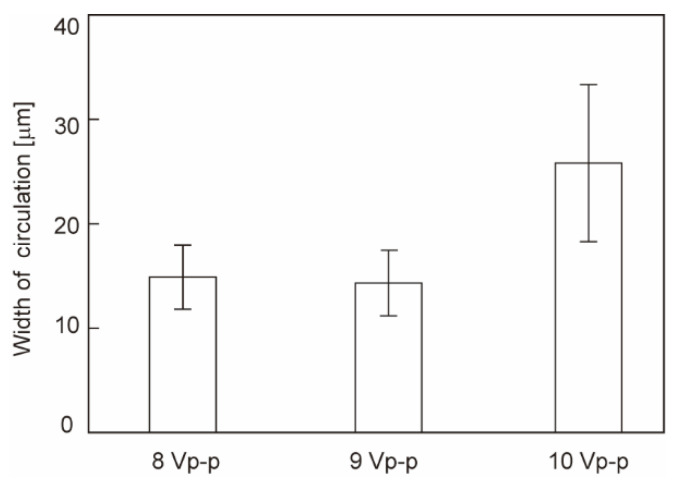
ACEO flow visualized by fluorescent microbeads. The width of circulation indicates the distance of movement of a microbead projected onto a horizontal plane. N = 4. Error bars show the standard deviation.

The electric field has a component parallel to the electrode surface, which moves ions in the solution along the electrode. The movement brings the flow of the solution resulting from its viscosity. The direction of the flow is independent of the electrical polarity of the applied voltage; therefore, the flow is stably induced even in AC voltage applications. The velocity *v* of the flow induced by the ACEO at a distance *x* from the electrode edge can be described as

(1) $$v=\frac{1}{8}\frac{\varepsilon V_{0}{}^{2}\Omega^{2}}{\eta x{\left(1+\Omega^{2}\right)}^{2}},$$

where $V_{0}$, ε, and η are the applied voltage, permittivity, and dynamic viscosity of the fluid, respectively, and Ω is

(2) $$\Omega =\omega \frac{\varepsilon }{\sigma }\frac{\pi }{2}x\kappa ,$$

where ω, σ, and κ are the angular frequency of the electric field, conductivity, and reciprocal Debye length, respectively [23]. Equation (1) shows that the velocity depends on the amplitude and frequency of the applied voltage. The velocity at the edge was the highest, increasing in accordance with the voltage. In our setup, the average velocity of the microparticles in the circulating flow was in the range of 15–23 μm/s. The width of the flow area increased from 15 to 27 μm (Figure 3), where the flow covered 25–45% of the electrode surface in accordance with the increase in voltage from 8 to 10 V_p-p_ at 10 kHz. This result suggests that a higher voltage results in an effective ACEO flow area at the electrode surface, enhancing the binding of molecules.

### 3.2. Enhancement of Protein Binding

We verified the feasibility of using ACEO in protein detection experiments using IgG as an analyte. The operation of anti-IgG immobilization through IgG detection requires a long time in batch experiments (typically ~1 h). Voltage application during the entire operation potentially changes the electrode conditions, resulting in failure to acquire the SPR sensorgram. Consequently, we limited the voltage application in ligand immobilization or analyte detection with a voltage setting of 10 V and 10 kHz. Under these conditions, ACEO was successfully induced, as illustrated in Figure 3. 

**Figure 4:**
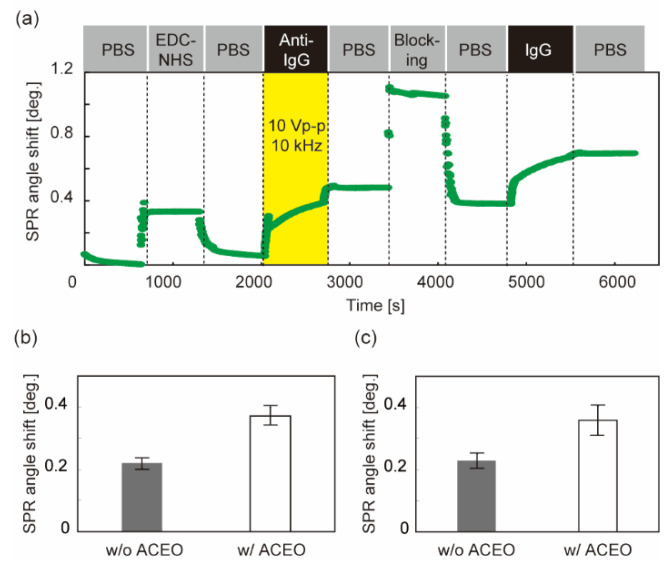
Detection of IgG molecules with ACEO-assisted SPR sensing. Sinusoidal voltage of 10 V_p-p_ and 10 kHz was applied during antibody immobilization. (**a**) SPR sensorgram (time course of the SPR angle shift) from antibody immobilization through IgG detection. The time window indicated by yellow shows the duration of voltage application. PBS: Washing process with 10 mM PBS. EDC-NHS: Immobilization of cross-linkers using EDC–NHS solution. Anti-IgG: Immobilization of antibodies that bind specifically to IgG molecules. Blocking: Application of ethanol–amine solution to block unreacted linker groups. IgG: Application of analyte (IgG). (**b**) SPR angle shift derived from the number of immobilized antibodies. (**c**) SPR angle shift derived from the analyte bindings. N = 4. Error bars show the standard deviation.

In the case of voltage application during anti-IgG immobilization, the SPR sensorgram was successfully obtained (Figure 4a). The shift in the SPR angle after washing with PBS indicates the number of bound molecules. The signals of both the ligand (anti-IgG) and analyte (IgG) increased compared to those without AC voltage (Figure 4b,c). The analyte signal was a factor of 1.5 higher than that without ACEO (Figure 4c) and is comparable to the increase in the ligand signal (Figure 4b). The results suggest that the ligands immobilized on the sensor chip increase with the contribution of ACEO, resulting in the enhancement of the binding of analyte molecules.

**Figure 5:**
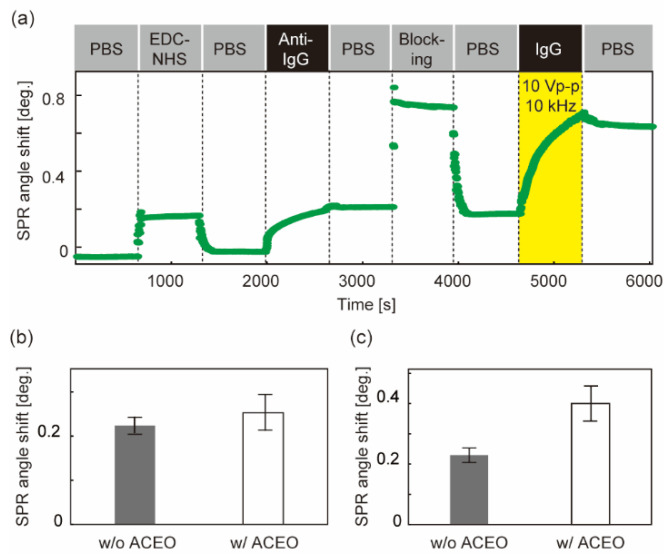
Detection of IgG molecules with ACEO-assisted SPR sensing: (**a**) SPR sensorgram. The style of the figure is the same as in Figure 4a; (**b**) SPR angle shift derived from the number of immobilized antibodies; (**c**) SPR angle shift derived from the analyte bindings. N = 4. Error bars show the standard deviation. Voltage was applied during analyte detection.

Voltage application during IgG detection also resulted in a successful SPR sensorgram (Figure 5a). The signal of the analyte (IgG) increased, whereas the ligand (anti-IgG) exhibited no significant difference, as expected (Figure 5b,c). The analyte signal increased by a factor of 1.7 above that without ACEO. These results suggest that the ACEO application successfully enhances the binding of the analyte to the ligand-immobilized surface.

**Figure 6:**
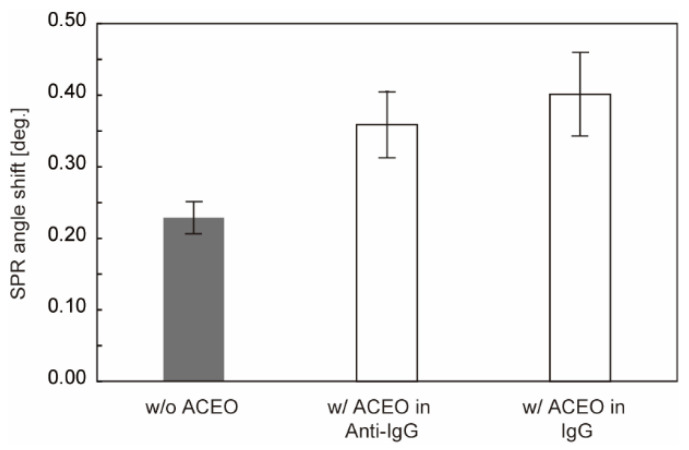
Comparison of SPR angle shift of analyte binding. w/o ACEO indicates the signal obtained from a conventional sensor chip without applying voltage. w/ACEO in Anti-IgG and w/ACEO in IgG indicate the signals obtained by the application of voltage in antibody-immobilization and analyte-binding steps, respectively (N = 4). Error bars show the standard deviation.

The results of the protein detection are summarized in Figure 6. Long-time application of voltage sometimes causes a change in the SPR curve to inhibit protein detection, which may be due to the electrolysis of Cr between the Au layer and glass. This led us to limit the voltage application in either the operation of ligand immobilization or analyte detection. However, the results demonstrate that both types of applications enhance analyte binding by increasing ligand immobilization and analyte binding to ligands, respectively. 

Signal enhancement will be useful for the detection of low-concentration proteins. Furthermore, ACEO produces another effect called “molecular shearing” [16]. The ACEO flow provides a shear force effectively on the sensor surface, which removes the nonspecific binding of molecules. For protein detection of a solution containing impurities (ex. serum [10,24], urine [25,26], and saliva [27,28,29]), the effect should improve the specificity along with the enhancement of protein detection reported here.

SPR sensing using direct current (DC) voltage has been reported in the literature [30,31], where the electrokinetic effect was employed to induce unidirectional flow for multi-sample transport [30] and to concentrate sample molecules prior to detection [31]. ACEO demonstrated here differently functions to enhance detection signal through the circulating flow on a sensor surface during the detection process, which can be compatible with the use of DC electrokinetic effect. The combination of these effects should bring a highly integrated SPR sensing system.

## 4. Conclusions

Protein detection using ACEO was demonstrated in this study. An SPR sensor chip with comb-shaped microelectrodes was fabricated using standard UV lithography to apply voltage to the ACEO. The chip allows us to use a standard Kretschmann-type SPR measurement system without an imaging function. We evaluated the application of AC voltage in ACEO and SPR sensing by changing the amplitude and frequency. We verified the ACEO-assisted increase in the binding of IgG molecules to the sensor surface. The results indicate that the amount of binding increases by a factor of 1.7 above that obtained using a conventional chip, demonstrating successful enhancement of the protein signal.

## Data Availability

The datasets are available from the corresponding author on reasonable request.
